# The Cardiac Center of Shisong Hospital: the first cardio-surgical center in West and Central Africa is inaugurated in Cameroon

**Published:** 2010-01-29

**Authors:** Appolonia Budzee, Jacques Cabral Tantchou Tchoumi, Jean Claude Ambassa, Alessandro Gimberti, Sylvia Cirri, Alessandro Frigiola, Gianfranco Butera

**Affiliations:** 1 Tertiary reverend Sister of St. Francis, Shisong, Cameroon,; 2 St. Elizabeth catholic general hospital, Shisong, cardiac centre, Cameroon,; 3 Policlinico San Donato, Department of paediatric cardiac surgery, San Donato, Italy

**Keywords:** Congenital heart diseases, acquired heart diseases, cardiac centre

## Special feature

The Italian charity organisation: “Associazione bambini cardiopatici nel mondo” (known in English as *Cardiac Children of the World*), was founded in Milan in 1994 by paediatric cardiac surgeon Alessandro Frigiola, and anaesthesiologist Silvia Cirri. The driving force behind the association is a passion for providing treatment and hope to disadvantaged children with congenital heart diseases in developing countries. The association has support from cardiac surgeons and cardiologists in Italy, as well as from famous cardio-surgical centres around the world, including the Mayo Clinic in Rochester, Minn; Great Ormond Street Children’s Hospital in London, England; and l’Hôpital des Enfants-Malades in Paris, France [1].

The Associazione Bambini Cardiopatici nel Mondo goals are to create autonomous centres and provide treatment in target countries. The association is presently involved in three main projects among which the Cameroon Project which was celebrated in 2001 in collaboration with the Tertiary Franciscan Sisters and the charity organisation Cuore Fratello. The project aim is to develop capacities for the diagnosis and treatment of congenital and acquired heart diseases in Cameroon. Three doctors, 3 technicians, and 10 nurses are being trained in Italy as part of the project human capacity development plan. During the last 2 years, children with simpler forms of cardiac disease have been treated in their own country by the Italian team and Cameroonian doctors in training. The most critical cases (about 40 children per year) are treated in San Donato (Italy). The inauguration of the Shisong Cardiac Centre took place in November 2009 [2, 3]. This historic event will significantly reduce the need to evacuate cardiac patients from Cameroon to Italy—an activity that Shisong Hospital has carried out since 2003.

Shisong Cardiac Centre is the only cardio-surgical Center in Central/West Africa, equipped with ultra modern technologies and prepared to offer a wide range of cardiology services including diagnosis and treatment of congenital heart defects, coronary artery disease, valvular heart disease and electrophysiology. It will handle non-invasive cardiology; that is, diagnostic testing for patients with suspected cardiac problems through tests such as electrocardiography, holter, stress test, three-dimensional colour, pulsed and continuous doppler- echocardiography; it will offer both diagnostic and interventional catheterism in a hemodynamic laboratory, implantation of permanent mono, bicameral pace makers and defibrillators. Open-heart surgeries with extracorporeal circulation will also be performed at the centre. The Project is jointly sponsored by the Tertiary Sisters of St. Francis, the Bambini Cardiopatici nel Mondo Association and Cuore Fratello Association in Milan (Italy) for an overall cost of 4 billion FCFA (800 millions USD).

The planning for inauguration went far beyond the spectrum of Shisong Hospital to involve all the Tertiary Sisters of St. Francis in Cameroon, Nigeria and the Republic of Central Africa; and an equal concern of the Bishop of Kumbo, Mgr. George Nkuo. The collaboration with the Senior Divisional Officer for Bui, Mr. Panjuouno Daniel and all the civil administrators of Bui, together with the Mayor of Kumbo Council, Mr. Donatus Njong, was phenomenal, a pace setter, indeed! At the divisional level Commissions were set to cater for various aspects such as road maintenance in town, accommodation, coordination, security, decoration and entertainment; and these set the actual celebration running at a grander scale!

“Even if the Prime Minister, the Minister, the Ambassadors and the Italian delegation were not to be present, we would still have a reason to celebrate this gift that has been brought to our environment by the Catholic Church through the Tertiary Sisters of St. Francis,” said the SDO of Kumbo at a preparatory meeting, which set everyone on their toes. The Cardiac Center was inaugurated by the Minister of Public Health, His Excellency Andre Mama Fouda, who stood as the personal representative of Cameroon’s Head of State. He proclaimed the Cardiac Center as the national reference Center, to the high applause of the multitude and promised subventions and other forms of reinforcement.

In the inaugural high Mass presided by his Eminence Cardinal Tumi Wirghan, he lauded Shisong Hospital for its excellent healthcare practice over the years and warned the medical staff against the encroaching hostile climate and lack of respect for life, and other gross deviations from the social teachings of the Church, threatening African today. In the same vein, Bishop George Nkuo, in his welcome address, invited all to pray and added that the facility in Shisong Hospital would give the Tertiary Sisters of St. Francis the opportunity to expand the scope and quality of their service.

Sr. Jethro Nkenglefac, Manager of the Cardiac Center, says she is aware of some major challenges involved in running a Cardiac Center, and therefore, is prepared to put up appropriate strategies for success. While thanking the Cameroon Government and its people for the support offered during its construction, she earnestly appealed to the public to make good use of the facility, to refer it to others within and beyond Cameroon, and to contribute in establishing the solidarity health scheme that would enable every person to benefit from specialized facility irrespective of social, racial or economic status.

“The Shisong-Bamenda and the Shisong-Foumban roads are not good for our patients,” said Sr. Euphrasia Yuh, Provincial Superior of the Tertiary Sisters of St. Francis, as she pleaded for the support of Cameroonian Government. The question of roads and electricity was common in the addresses of the Mayor of Kumbo, the Italian Ambassador to Cameroon, the Major Superior of the Capuchins, and those of the two Partner-Associations. And, in fact, an 83-person delegation was getting ready to pour from Italy and France unto the Cameroonian soil, consisting of cardiac surgeons, specialized nurses, politicians, donors, journalists, and members of the two Associations. Professor Alessandro Frigiola, pediatric and adult cardiac surgeon and Founding President of Bambini Cardiopatici nel Mondo, visited Cameroon for the first time and symbolically operated the first two patients in the new theater on November 20 2009. Subsequent surgeries will be performed by Dr. Alessandro Giamberti and Dr. Charles Mvondo.

Don Claudio Maggioni, the Milan-based diocesan priest who first dreamed of the Cardiac Center in a developing world, said he was humbled to witness the extent to which the mustard seed had spread and the number of birds that take shelter in its branches. Marking the inauguration was the medical conference under the theme “pediatric and adult cardiology”, which brought together guest speakers from Italy, France, and Cameroon. Participants involved health personnel from most of the health institutions in Bui and beyond.
